# Nursing rights in comics: educational technological innovation report

**DOI:** 10.1590/0034-7167-2023-0438

**Published:** 2024-03-18

**Authors:** Odilon Vieira Santos, Meiry Fernanda Pinto Okuno, Paula Hino, Mônica Taminato, Dulce Aparecida Barbosa, Hugo Fernandes

**Affiliations:** I Universidade Federal de São Paulo. São Paulo, São Paulo, Brazil

**Keywords:** Ethics, Nursing, Technological Development and Innovation Projects, Graphic Novel, Education, Nursing, Nursing., Ética en Enfermería, Proyectos de Desarrollo Tecnológico e Innovación, Novela Gráfica, Educación en Enfermería, Enfermería., Ética em Enfermagem, Projetos de Desenvolvimento Tecnológico e Inovação, História em Quadrinhos, Educação em Enfermagem, Enfermagem.

## Abstract

**Objectives:**

to report an educational technology construction on nursing professionals’ rights.

**Methods:**

an experience report on educational technology construction during the crediting of university extension hours in an undergraduate nursing course at a Brazilian public university, between March and June 2023. The Deming cycle was used as a procedural method.

**Results:**

four meetings were held between students and extension workers. Eight comic books were produced based on the Code of Ethics for Nurses, addressing professional autonomy, fair remuneration, risk-free work, denial of exposure in the media and others. The Deming cycle proved to be an important strategy for constructing products.

**Conclusions:**

nursing professionals’ rights must be discussed and improved. Educational technologies, such as comic books, provide playful and reflective learning.

## RESUMO

**Objetivos:**

relatar a construção de uma tecnologia educacional sobre os direitos dos profissionais de enfermagem.

**Métodos:**

relato de experiência sobre a construção de tecnologia educacional durante a creditação de horas de extensão universitária em uma disciplina da graduação em enfermagem de uma universidade pública brasileira, entre março e junho de 2023. Utilizou-se o ciclo de Deming como método processual.

**Resultados:**

foram realizados quatro encontros entre estudantes e extensionistas. Oito histórias em quadrinhos foram produzidas pautadas no Código de Ética dos Profissionais de Enfermagem abordando autonomia profissional, remuneração justa, trabalho sem riscos, negação à exposição em mídias e outros. O ciclo de Deming mostrou-se importante estratégia para construção dos produtos.

**Conclusões:**

os direitos dos profissionais de enfermagem devem ser discutidos e aprimorados. Tecnologias educacionais, como histórias em quadrinhos, proporcionam aprendizagem lúdica e reflexiva.

## RESUMEN

**Objetivos:**

informar la construcción de una tecnología educativa sobre los derechos de los profesionales de enfermería.

**Métodos:**

relato de experiencia sobre la construcción de tecnología educativa durante la acreditación de horas de extensión universitaria en una carrera de pregrado en enfermería en una universidad pública brasileña, entre marzo y junio de 2023. Se utilizó el ciclo de Deming como método procesal.

**Resultados:**

se realizaron cuatro reuniones entre estudiantes y extensionistas. Se produjeron ocho historietas basadas en el Código de Ética para los Profesionales de Enfermería, abordando la autonomía profesional, la remuneración justa, el trabajo libre de riesgos, la negación de la exposición en los medios de comunicación y otros. El ciclo de Deming demostró ser una estrategia importante para la construcción de productos.

**Conclusiones:**

los derechos de los profesionales de enfermería deben ser discutidos y mejorados. Las tecnologías educativas, como los cómics, proporcionan un aprendizaje lúdico y reflexivo.

## INTRODUCTION

Nursing rights are an important issue both in the world and in Brazil, aiming to guarantee adequate working conditions, fair remuneration, safety and legal protection for nursing professionals^(1)^. Internationally, nursing rights have been recognized and defended by organizations such as the World Health Organization (WHO) and the International Council of Nurses (ICN). The WHO has emphasized the need to invest in the nursing workforce, promoting better working conditions and profession appreciation. The ICN, in turn, works to establish ethical standards and safe professional practices, in addition to advocating for nurses rights around the world^(2-3)^.

In Brazil, nursing is regulated by Professional Practice Law (Law 7,498/1986) and the Code of Ethics for Nurses. These laws guarantee the legal exercise of the profession, establishing the duties of different nursing professionals (nurses, nursing technicians and assistants) and determining the rights and duties of professionals^(4)^.

Chapter I “Rights” of the Code of Ethics for Nurses in Brazil establishes the fundamental rights of professionals in this area. In this chapter, the rights to dignity, freedom, security, privacy, respect, professional appreciation and participation in decisions about their practice are highlighted. These rights not only promote nursing professionals’ well-being, but also contribute to the quality of care provided to patients, establishing solid ethical and human foundations for nursing practice^(4)^.

Nursing professionals are usually charged with their duties during the exercise of their profession; however, they rarely have appropriate working conditions or are unaware of their rights in depth. Such aspects can affect their well-being and even their general and work health^(2)^.

One of the strategies for guaranteeing professional rights in health is the promotion of ongoing education on workers’ practical or daily aspects. This education can be carried out in different ways, including playful and enjoyable strategies. This form of teaching can be called image literature, which explores using visual resources and particularities of everyday language to construct knowledge. One of its strategies is the comic book, which has temporal dimensions (through a linear sequence of images) and spatial dimensions (through the application of the logic of the elements and narratives that make up the scripts). Image literature is an entertainment activity, with high potential intersection with teaching and learning for people of different age groups, including adults, considering that it allows conveying knowledge and information in a pleasurable way.

Comic books are a visually attractive and engaging resource for conveying complex information in an accessible and interesting way. They combine text and images concisely, which makes it easier for nursing professionals to understand and retain the content. One of its main advantages is its ability to convey information in an engaging way. Characters and plots can arouse readers’ interest, making the learning process more enjoyable and motivating^(5)^. This is especially relevant in continuing health education, where the review of concepts and information must be constant, but can often be perceived as monotonous and uninteresting^(5-6)^.

Additionally, comic books can address a wide variety of health-related themes and scenarios, as they allow to explore real clinical situations, ethical dilemmas, teamwork challenges, patient communication issues, and much more. Thus, comic books can be adapted to different areas of nursing, meeting the specific needs of each profession or specialty^(7)^.

Studies also indicate that comic books can be easily shared and disseminated, whether in physical or digital format, allowing readers to access the material in a convenient and flexible way. This is especially relevant in times of advanced technology, where quick and easy access to knowledge is crucial. Therefore, using comics for health training or continuing education offers an innovative and effective approach to conveying information, making learning more effective^(6-7)^.

Faced with the demand for the inclusion of university extension in the curricular matrices of higher education courses in Brazil, the authors of this study identified in comic books the possibility of forming part of a training itinerary for nursing graduates during a curricular unit focused on ethics and to professional legislation, especially addressing the rights and struggles of the professional category, with the possibility of giving back to society, especially to already trained professionals so that they fight for the preservation of their rights and guarantees.

## OBJECTIVES

To report the process of educational technology construction on nursing professionals’ rights.

## METHOD

This is an experience report on educational technology construction (comic books) on nursing professionals’ rights by members of an extension project and nursing students during the implementation of “curricularization” of university extension in a curricular unit of undergraduate nursing at the *Universidade Federal de São Paulo* (UNIFESP) between March and June 2023. The term “curricularization” has been adopted in universities to mention the accreditation of extension actions in undergraduate curricular units, in compliance with goal 12.7 of the Brazilian National Education Plan, which provides for the implementation of extension actions in the Pedagogical Course Project in at least 10% of its total workload^(8)^.

The project mentioned is called *Jano - Cultura de Paz*, and its main objective is to articulate and implement university extension actions aimed at achieving the premises of a culture of peace established by the United Nations Educational, Scientific and Cultural Organization (UNESCO) and with the 16^th^ Sustainable Development Goal (SDG) established by the United Nations (UN) in the 2030 agenda, which seeks to achieve peace, justice and effective institutions. The project activities are developed on the UNIFESP São Paulo *campus*, and involve professors, undergraduate students, graduate students, professionals and people from the non-academic community. One of the curricular units that implemented the crediting of extension hours linked to the Jano project was Legislation and Ethics, which teaches the deontological bases of nursing.

The product of this report was created through the interactive process and product management method called the Deming cycle or PDSA, related to the verbs To Plan, To Do, To Study and To Act. The use of this method is justified for promoting experimentation, systematic learning and adaptation, allowing knowledge construction to become more active, meaningful and orderly throughout its process. Furthermore, this method is widely used in nursing and other areas of health^(9)^, proving to be an interesting tool for constructing collective products.

As this is an experience report, consideration and approval by the Research Ethics Committee were not necessary, as provided for in Resolution 466/2012 of the Brazilian National Health Council.

## RESULTS

It took two months for the complete Deming cycle and four meetings among students, extension workers and extension project coordinators. In the To Plan stage, participants divided themselves into working groups, seeking sources of inspiration (such as comic books, anime and other forms of existing comic books) and carrying out an in-depth reading of Resolution 564 of 2017 of the Federal Nursing Council (COFEN), which instituted a review of the Code of Ethics for Nurses, which served as the main theoretical framework for the project. The search for supplementary materials, such as books and articles on the topic, was encouraged. They also defined the target audience for the products: nursing professionals and students at secondary and higher education levels for ongoing education on their rights. The To Do stage included the precursor idea and title construction and story script elaboration. Still at this stage, students chose the applications necessary to create the illustrations, and using resources easily available for computers or smartphones was recommended. The applications used were Comic Creator^®^, Comica^®^, Comic Book^®^ and Canva^®^, all in free versions. The story outline was prepared, including naming characters and setting roles.

**Figure 1:**
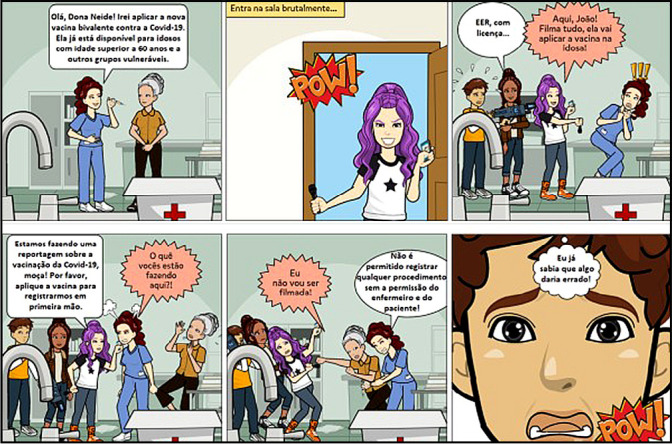
Image present in the collection “Fight and Respect: Comics about Ethics and the Fight for Nursing Rights”, São Paulo, São Paulo, Brazil 2023

In the next stage, To Check, the preliminary products were presented to lay people, such as members of the community around UNIFESP, participants in the Jano project, in order to obtain suggestions for improvements in images, texts and material content. This process allowed for in-depth information and greater reliability in the reach of comic books to the target audience. The review especially included reducing the content of the narratives, as statements were identified as long and complex in some materials. Furthermore, two materials were adjusted so that the story had an end, as they gave the impression that there would be a sequel, a fact that was unfeasible at the time. Finally, in the To Act stage, comic books were completed, and their definitive version was sent by the authors to the Ethics and Legislation curricular unit coordinators and the Jano extension project. The final products were organized in a collection called “Fight and Respect: Comics about Ethics and the Fight for Nursing Rights”, as illustrated in Figure 1.

The total number of people involved was 80, including two professors, two graduate students, four community members (health professionals), 62 undergraduate nursing students, five extension workers and one extension project scholarship holder. The final products received a printed version, with a circulation of 50 copies, which were part of an exhibition fair at *Escola Paulista de Enfermagem*, in addition to being distributed free of charge to nursing professionals at *Hospital São Paulo.* The educational technology created proved to be very attractive, playful and easy to understand, allowing wide use among secondary and higher education professionals.

It should be noted that the digital figures used in the comics were already images from the chosen applications, with permission for use and dissemination, without copyright restrictions. A single product was illustrated manually by one of the authors who had greater illustrative skills.

To ensure product authenticity and enable their wide use, the authors obtained a Creative Commons International 4.0 license registration, which allows public attribution and free use. Furthermore, in order to preserve intellectual production, the materials were deposited in the UNIFESP Institutional Repository, accessible through the following address: https://repositorio.unifesp.br/11600/68469.

## DISCUSSION

Nursing workers play a fundamental role in the health care sector, being responsible for providing direct care to patients, promoting health, preventing illnesses and assisting in the recovery process. Despite the importance of these workers, they often face precarious working conditions, long hours, lack of professional appreciation and recognition, low wages and lack of safety in the work environment. These issues have historically motivated nursing professionals to mobilize in search of better working conditions and guarantee their rights^(3,10)^.

The Code of Ethics for Nurses, used as the main framework for creating the comics in this report, establishes the profession rights, guiding the ethical exercise and guaranteeing the quality of care provided. These rights ensure professional autonomy, allowing nurses to act independently, based on scientific and technical knowledge. Furthermore, professional development is highlighted, covering fair remuneration, adequate working conditions and professional development. Rights that were also highlighted in the comics were respect for privacy and ensuring patient confidentiality, highlighting the importance of intimacy and protecting confidential information obtained during care^(4)^.

Active participation in health decisions and policies was a fundamental nursing right highlighted in the stories, allowing professionals to contribute their technical knowledge and expertise. Authors mention that this participation takes place through active presence in committees, councils and forums that discuss issues related to health and public policies^(2-3)^.

Workplace safety and evidence-based practices were also highlighted in the comics created. Both conditions are aligned with the UN SDG, which seeks decent work and sustainable growth, where “full employment” must be respectful, inclusive and safe for all^(10)^. This includes ensuring access to adequate personal protective equipment, receiving training in occupational health and safety as well as implementing preventive actions to avoid work-related accidents and illnesses. At the same time, acting based on scientific evidence is a right that strengthens nursing practice, ensuring that clinical decisions are based on up-to-date knowledge and best practices. Nurses have the right to constantly seek knowledge, participate in continuing education programs and use guidelines and protocols based on scientific evidence^(1-3)^. By combining these rights, it is recognized that a safe and healthy work environment provides the necessary conditions for nursing professionals to seek, apply and improve scientific knowledge, resulting in evidence-based quality care that promotes patient well-being^(4)^.

Furthermore, the refusal to carry out practices that are unethical, putting the health and safety of patients at risk, or that are illegal, was highlighted in the products. It is interesting to mention that the Code of Ethics for Nurses ensures that professionals must have the autonomy to question and reject inappropriate conduct, basing their actions on principles such as justice, beneficence and respect for human dignity^(2,4)^.

Comic book creation was an effective educational tool that helped nursing students understand the profession rights. This playful and engaging approach facilitated the assimilation of concepts and encouraged students’ active participation in the learning process^(5,7)^. For trained and working professionals, comic books can promote a better understanding of the rights they have in the exercise of their profession, because the combination of visual and narrative elements to address aspects such as professional autonomy, participation in health policies, patient confidentiality, workplace safety and evidence-based practice generates emotional involvement with characters and projection about their own practices, facilitating assimilation and internalization of concepts. Furthermore, comic books can provide an interactive form of learning, stimulating discussions and exchange of experiences among professionals^(6)^.

The use of Deming cycle or PDSA was very useful for the proposals. Its systematic structure stands out as an advantage, which provided clear and organized guidance for learning^(9)^. It guided students through well-defined stages, from planning to action, promoting a methodical approach. Furthermore, the cycle emphasized practical and dialogue-based learning, allowing students to apply what they studied in simulated situations (comics). Another advantage noted was the emphasis on continuous improvement. The PDSA cycle encouraged interaction, allowing students to experiment with different approaches, analyze results, identify knowledge gaps, and make adjustments to their subsequent actions. This promoted more effective learning over the proposed time, encouraging them to improve their products. As a disadvantage, it should be noted that implementation required considerable time and effort by students, as each stage required a different logic. Furthermore, the effective application of this work method required close supervision of the educators involved and the coordination of the extension project to avoid dispersions and optimize time in each phase.

Finally, products constructed through extension credit in undergraduate education promote greater meaning for both students and the community involved, not only returning knowledge to society quickly, but actively involving it in actions that will somehow affect it. Thus, the “curricularization” of university extension has proven to be a viable way of bringing the university closer to the community in a participatory and productive way^(8)^.

### Study limitations

Considering that this is an experience report on educational technology construction on nursing professionals’ rights, the products produced did not undergo content validity with experts, suggesting such activity in the future. Another limitation is that the materials were only produced in Portuguese, with future adaptations to other languages.

### Contributions to science and nursing

The struggles to guarantee nursing rights should never be abandoned, as they not only ensure good conditions for the profession, but also allow for constant improvements. Thus, playful conveying through comics on this aspect promotes enjoyable learning and allows those who have already graduated to maintain ongoing education on one of the axes of the Code of Ethics for Nurses.

### CONCLUSIONS

Eight comic strips were created during the implementation of crediting hours for an extension project in an undergraduate nursing course on ethics and professional legislation. The students and extensionists involved used Resolution 564 of 2017 of COFEN as the main theoretical framework to prepare the products. The PDSA cycle was extremely timely, as it allowed construction to be systematized in an organized and continuous manner, in addition to allowing the use of interpersonal skills of those involved, such as teamwork, communication and creativity. Thus, the tool shows promise for other scenarios where it is necessary to construct educational products in a participatory and socialized way.

Finally, the participation in extension projects in curricular units or undergraduate subjects transcends the obligation provided for in legislation, standing out as a permanent resource for improving university education and reflecting directly and indirectly on society.

## Data Availability

“*Coletânea: Luta e respeito: Histórias em quadrinhos sobre ética e luta pelos Direitos da Enfermagem*”, https://repositorio.unifesp.br/11600/68469, UNIFESP Repository, Metadata.
